# Network-based approaches to quantify multicellular development

**DOI:** 10.1098/rsif.2017.0484

**Published:** 2017-10-11

**Authors:** Matthew D. B. Jackson, Salva Duran-Nebreda, George W. Bassel

**Affiliations:** School of Biosciences, University of Birmingham, Birmingham B15 2TT, UK

**Keywords:** development, network science, self-organization, structure–function, complexity, multicellularity

## Abstract

Multicellularity and cellular cooperation confer novel functions on organs following a structure–function relationship. How regulated cell migration, division and differentiation events generate cellular arrangements has been investigated, providing insight into the regulation of genetically encoded patterning processes. Much less is known about the higher-order properties of cellular organization within organs, and how their functional coordination through global spatial relations shape and constrain organ function. Key questions to be addressed include: why are cells organized in the way they are? What is the significance of the patterns of cellular organization selected for by evolution? What other configurations are possible? These may be addressed through a combination of global cellular interaction mapping and network science to uncover the relationship between organ structure and function. Using this approach, global cellular organization can be discretized and analysed, providing a quantitative framework to explore developmental processes. Each of the local and global properties of integrated multicellular systems can be analysed and compared across different tissues and models in discrete terms. Advances in high-resolution microscopy and image analysis continue to make cellular interaction mapping possible in an increasing variety of biological systems and tissues, broadening the further potential application of this approach. Understanding the higher-order properties of complex cellular assemblies provides the opportunity to explore the evolution and constraints of cell organization, establishing structure–function relationships that can guide future organ design.

## 1. Introduction

The advent of multicellularity represents one of the major evolutionary transitions [1], arising independently and persisting at least 25 times during the evolution of life in our biosphere [2]. Multicellular systems are characterized by functional division of labour across members of a consortia, making use of diversification as a means of overcoming environmental constraints [1,3–5]. However, the benefits of cellular cooperation must also be balanced with the increased costs and risks associated with conflicts and cheats, leading to a need for optimization [4], self-policing strategies and the emergence of identity [6,7].

Structure–function relationships have been described previously at an organ level [8], and these principles are proposed to scale down to a cellular level [9]. Novel cellular arrangements can confer novel functions to organs, enabling organisms to fill ecological niches previously left vacant. The lack of a quantitative framework to capture, analyse and compare the organization of organs at a cellular level limits the ability to uncover the functional role of observed structures.

While research into individual components of complex biological systems is fundamental to our understanding of life, understanding how these components come together to form a coherent and functional system is a distinct concept. Assemblies of cells represent templates upon which genetic developmental programmes can act, giving rise to complexity for free [10] upon which further complexity might be built. Genetically encoded mechanisms lead to the creation and regulated organization of these cellular assemblies; they therefore create, and in turn operate, within cellular networks. This represents an integrated dynamical system across the cellular and molecular levels of description.

Efforts to understand the genetic basis of patterning have typically been directed to the study of signalling and differentiation processes, physical mechanisms related to the movement of cells [11,12], the regulation of local cell divisions [13,14] and programmed cell death [15]. While fundamental and informative, the contextualization of these confined events into the global context of multicellular organs, and the emergent properties of complex multicellular assemblies, remains limited due to the qualitative and local nature of these descriptions. Numerous outstanding questions surrounding higher-order principles of organ design and cohesion in diverse systems persist.

To address these fundamental questions of complex organ design, there remains a need to be able to capture, quantify and characterize global cellular organization and its properties. Here, we discuss a framework that strives to achieve this.

## 2. Organs and tissues as networks of cells

**Figure 1.**
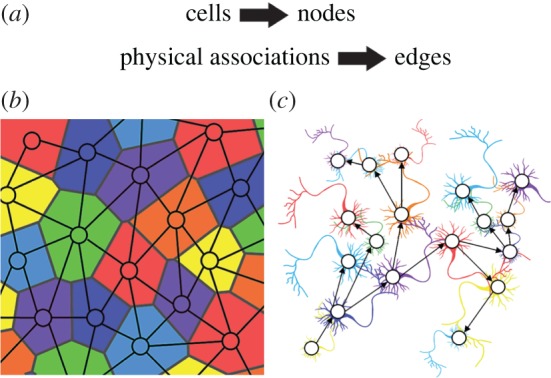
Discretization and abstraction of cellular organization into networks. (*a*) Cellular interaction mapping leads to the generation of networks where the nodes represent cells and edges their physical interactions. (*b*) A diagram of a cell interaction network typical of epithelial tissues in plants and animals. (*c*) A diagram of a part of a directed network of neuronal interactions. Information flows from the axon of a neuron to the dendrites of connected neurons giving the edges directions.

Understanding the structural basis of cellular organization can be achieved by mapping cellular interactions. Cells within multicellular organs interact physically and chemically to create coherent systems. In light of these relationships, similarly to how road and rail transport networks connect places of interest, organs may be viewed as complex systems of interacting cells. Here, cells are represented by nodes, and their physical associations by edges (figure 1*a*,*b*). The ability to capture and abstract cellular connectivity into networks allows their analysis using network science [16,17].

## 3. The origins of cellular interaction mapping

The mapping of cellular associations in complex organs was first explored in the field of neuroscience. Ramon y Cajal made seminal observations relating to the connectivity of neurons [18]. Nervous tissue was stained and examined using light microscopy to establish the proximal cell associations, which were carefully hand drawn to create ‘wiring diagrams’. This work set a key precedent for the future analysis of cellular associations in neuroscience.

The subsequent work of the laboratory of Sydney Brenner used serial transmission electron microscopy (TEM) sections to map all the neuronal interactions, or the ‘connectome’, within the worm *Caenorhabditis elegans* [19]. The relationships between cells were represented as a directed network of nervous connectivity (figure 1*c*). This represented the first comprehensive description of interactions within a given cell type, and has gone on to serve as a powerful template that has guided hypothesis generation and analysis of this complex system of cells [20]. Neuronal connectivity mapping has persisted and become increasingly more ambitious with time, with ongoing projects seeking to map more complex nervous systems, and are discussed further below [21,22].

We propose that mapping cellular associations may also be applied to understanding cellular complexity and organ function in diverse biological systems outside of the nervous system, and that this approach can address questions of central significance to developmental biology and the origins of multicellularity [23].

## 4. Structural and functional networks

In the analysis of multicellular structures, an important distinction is drawn between how cells are physically connected and how information in fact moves between these cells. These have been termed structural and functional networks, respectively [21].

**Figure 2.**
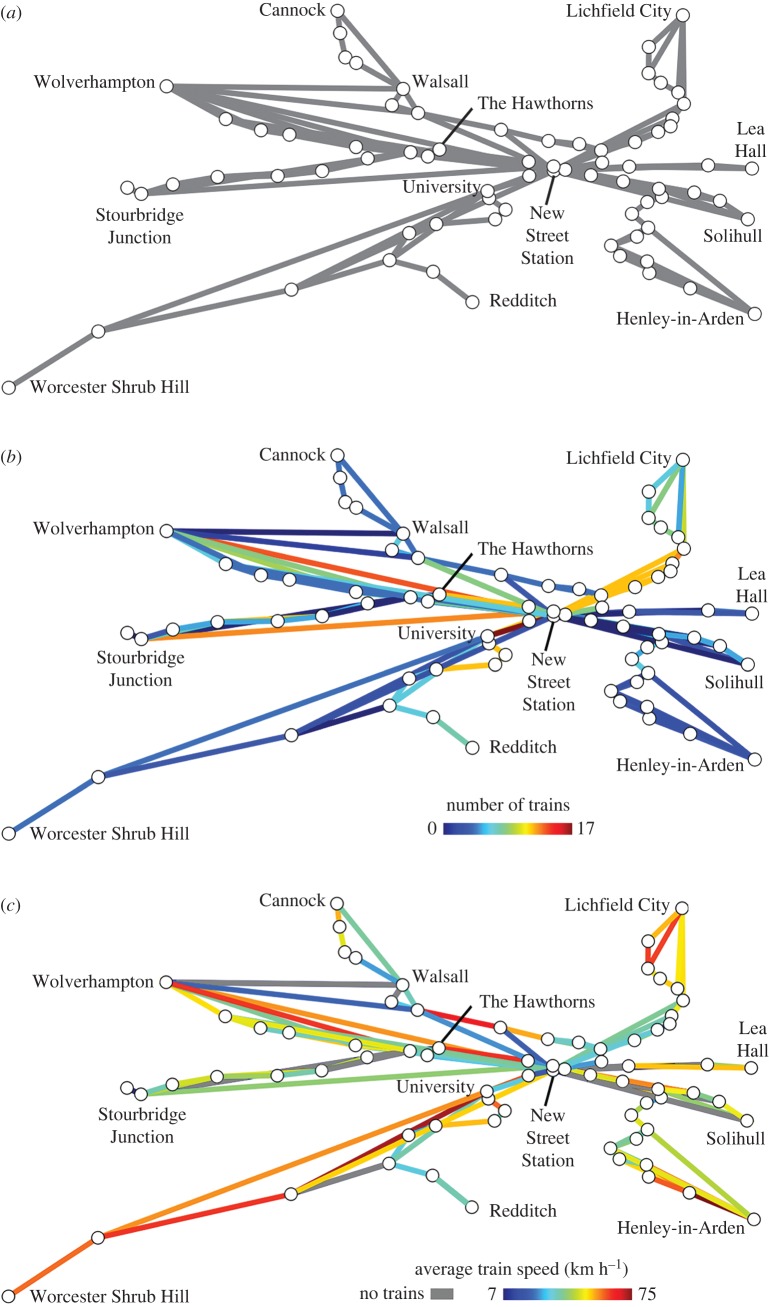
Structural and functional networks of the Birmingham, UK, rail system. (*a*) The structural network of the Birmingham rail system, showing possible routes (edges) that can be taken between stations (nodes). (*b*) Functional annotation of the rail network, where edges are false coloured by the frequency of trains running between stations between 08.00 and 10.00 on weekdays. (*c*) Functional annotation of the rail network, where edges are false coloured by the average speed of trains between 08.00 and 10.00 during a weekday.

Structural networks describe the physical associations between cells, and the possible routes of information movement through an organ [21] (figure 2*a*). This is analogous to a road or train map which shows all the possible paths a traveller could take.

Functional networks describe where information is observed to move. This is equivalent to a train schedule which describes the frequency (figure 2*b*) and speed (figure 2*c*) of travel across the rail network, describing a behaviour of the system. Structural networks serve as templates upon which functional events occur. These two dimensions of the system are intricately linked and shape and constrain one another. Following the establishment of structural templates of organs, their annotation with further functional data enables the creation of multidimensional views of the molecular dynamics and their topological relationships within organs. Approaches to achieve this are discussed below.

The nature of functional mobile information remains open to interpretation. We propose this to include any non-cell autonomous signal that plays an instructive functional role across a multicellular system. In the case of neuronal associations, neurotransmitters eliciting responses in adjacent cells represent a clear example. In other organs, information can take different forms, including small molecules, proteins or extracellular interactions between proteins in adjacent cells [24,25]. This transfer of information can occur either through extracellular spaces or through cytosolic connections between adjacent cells [26,27]. In plants, whole proteins, mRNAs and miRNAs have all been observed to move from one cell to the next [28,29]. Mechanical interactions between adjacent cells may also be considered a form of non-cell autonomous information in light of the instructive nature of these signals [30]. In each case, this information is received by the cells (nodes) of the network, and transmitted through intercellular interactions (edges).

## 5. Analysis of cellular interaction networks

The abstraction and discretization of cellular associations within organs into networks provides a means to quantitatively analyse their properties. Towards this, an appropriate analytical framework is required. Network science and the tools developed by this scientific community are capable of fulfilling this analytical task [16,17,21].

Two different scales may be topologically examined in cellular connectivity networks: local and global, depending on the centrality calculation performed.

**Figure 3.**
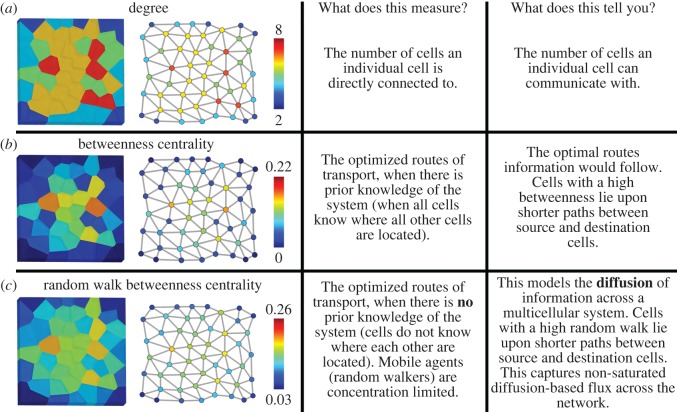
Topological features of virtually generated planar cellular connectivity network. (*a*) Degree false coloured on the virtual tissue and the corresponding networks according to the scale provided. (*b*) Same as (*a*) with betweenness centrality. (*c*) Same as (*a*) with random walk centrality.

The simplest local property of a cell in an organ is the number of immediate neighbours a cell has. This is called node degree, and has been successfully used in previous studies examining the organization of epithelial tissues using planar cell connectivity networks [31,32] (figure 3*a*). Degree is an informative feature describing the local context of a cell; however, it does not provide information relating to the higher-order properties of the organ and how an individual fits into a broader context.

In light of the geometric constraints of cells in physical space and their packing within organs, cellular connectivity networks can be considered to be spatially embedded [33]. These may be planar in the case of cellular monolayers such as the *Drosophila* wing disc, or a three-dimensional (3D) lattice as in more complex organs such as the brain. This geometrically constraining property means the ability for information to optimally traverse the multicellular network will be defined by the topological features of cell configurations. The identification of optimized (and counter-optimized) routes through organs can be achieved through the analysis of path length [16,17]. The requirement for cohesion between cells in conferring organ function makes this a biologically relevant feature of these spatially embedded multicellular systems. To use the analogy of a city, degree describes who one lives beside, while path length analyses would let one know where they are located within a city, and the fastest routes to follow to get to any other location. Both represent distinct and important pieces of information depending on the biological question being addressed.

A range of network centrality measures have been developed to explore path length [16,17] (figure 3*b*,*c*). Betweenness centrality uses prior knowledge of a network to calculate the number of times a node lies on the shortest path between other nodes (figure 3*b*) [34]. This identifies cells which are ‘brokers’ having the ability to control the movement of information. This may provide insight into the optimization of established long-distance transport processes in organs.

Random walk betweenness centrality does not use prior knowledge of the network, and identifies shortest paths by measuring the number of times a random walker follows a given route between two chosen nodes (figure 3*c*) [35]. When nodes are found to be traversed more frequently than others, they are deemed to lie upon shorter paths. This is analogous to not having a map and iteratively choosing random trains until you reach a final destination. Done enough times, the best travel options are eventually identified. Cells which have a high random walk betweenness centrality are therefore topologically poised to experience a larger amount of information flux than other cells. This centrality is analogous to measuring diffusive processes across networks, which play an important role in diverse aspects in organ biology.

Each of these centralities can provide insight into the underlying behaviour of multicellular systems. To summarize these differences in simple terms, one could interpret the capture of a process by betweenness centrality as a system having prior knowledge of the global layout of cellular organization when regulating information flow. Feedback from destination to source could also achieve such optimization [36]. Random walk centrality captures the diffusion of a non-saturated mobile signal between randomly selected producer and receiver cells, analogous to the movement of a morphogen across a system.

These network science centralities exploring local connectivity through degree and global path length properties provide ways of revealing biologically relevant properties of multicellular organization in organs. There, however, remains scope for the development of additional centralities which capture the properties and constraints of the spatial embedding of cells in organs [37,38]. These, among other metrics, represent potential measures to explore the higher-order organization of cells in organs, and optimization of transport within complex cellular assemblies.

Additional network metrics beyond centrality may also be used to analyse cellular connectivity networks. For instance, the detection of communities or modules within networks has received quite some focus by the network science community [39]. This approach could enable the identification of functional modules and structures within organs. Several metrics and algorithms are available in this case, including hierarchical clustering and modularity maximization methods [40].

On the other hand, functional annotated networks may be analysed using these new layers of data, for instance exploring the properties of signal distributions or the movement of information across structural templates. This approach has been widely embraced by the field of neuroscience [21]. A recent, although contentious, example of this can be found in the integrated information metric used by Tononi [41].

There is no one-size-fits-all topological measurement for cellular interaction networks. Depending on the question being asked and the nature of the biological system, different combinations of topological analyses may be applied. It is worth noting that network size should be used to normalize data [42].

Another interesting aspect to consider when analysing many types of networks is how efficient they are in terms of facilitating or impeding movement across them. This might relate to the flow of information (like in the brain or the Internet), the flow of smart entities that can route themselves through optimal paths (like humans in the transport network) or the random walk of diffusible molecules through compartments (perhaps not unlike the movement of small molecules in a cellular connectome). To characterize efficiency in transport networks, several measurements are available, some of which are discussed below.

Global efficiency [43] concerns itself with the average distance between each pair of nodes within a network, a concept closely related to that of small worlds in social network science [44]. Networks might display much shorter average shortest path lengths if there are shortcuts which can greatly reduce average distances by connecting regions that otherwise would be far apart. This measure can be used to compare different networks [37,43], although special care needs to be taken when contrasting networks have different sizes or connectivity.

The counterpart of transport efficiency is transport robustness, also called local efficiency in some studies [43]. This metric relates to the ability of a network to continue to function with faulty components, and is computed by locally comparing the changes in average path lengths before and after the removal of edges. Displaying high transport robustness is also related to a high global clustering coefficient [45] or the frequency of triangle motifs.

Interestingly, under the constraint of preserving the number of nodes and edges, a trade-off between local and global efficiency has been demonstrated to be present [43]. On the one hand, spatially embedded and homogeneous systems (such as regular lattices) are known to be highly resistant to the loss of edges while displaying low global efficiencies. At the other side of the spectrum, random graphs contain shortcuts that reduce average path lengths and increase global efficiencies, but are not structured and thus suffer heavily under random faulty components. Such a trade-off relation can be coalesced into a pareto front [46,47]. Under this view, different systems can be compared and ranked according to their optimality in this trade-off. Such a value can be regarded as a proxy for fitness, creating a direct connection between the topological analysis of cellular configurations across genetic and evolutionary contexts.

## 6. Models of multicellularity

A variety of models describing multicellular systems have been generated and analysed to varying extents previously [48]. These models have generally sought to understand how generative genetic and mechanical rules give rise to pattern formation in diverse contexts and scales. These approaches have provided limited regard to the underlying organization or self-organization of cellular neighbourhoods and topologies, commonly making use of regular lattices instead. The comparison of the outputs of these models and observed biological tissues has been largely limited to qualitative comparisons.

The application of a network-based approach to understanding complex cellular organization in each biological and simulated system provides a framework in which to make quantitative comparisons, enabling more concrete statements to be made about the similarity of the models to living systems. This may also provide an avenue for undiscovered mechanisms of pattern formation to be uncovered.

**Figure 4.**
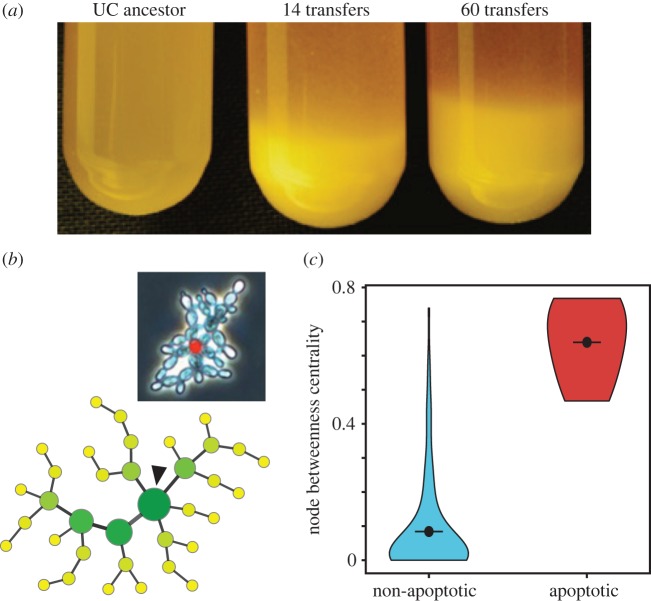
Using a network framework to understand multicellular systems. (*a*) Experimental evolution of multicellularity by Ratcliff *et al.* [49]. As the number of sequential transfers increases, a unicellular (UC) yeast population evolves to become multicellular (MC) with higher sedimentation speeds. (*b*) Newly evolved phenotype called ‘snowflake’ (inset), with ensembles of cells remaining physically attached due to defective fission. Cells undergoing apoptosis are stained red. These events will split up the aggregate, leading to further growth. Below, the actual network underlying this particular ‘snowflake’, with node betweenness centrality depicted in shades of green and an arrow pointing at the sole apoptotic cell in this ‘snowflake’. (*c*) Violin plot of betweenness centrality, separating the cells into apoptotic and non-apoptotic. Dots and lines represent the means of each distribution, three aggregates and 118 cells.

To illustrate the ability of this analytical framework to identify biologically relevant features in complex cellular assemblies, we performed a meta-analysis of the data generated in [49]. In this work, the authors demonstrated that cellular assemblies with multicellular traits could be obtained by means of artificially selecting faster gravitational sedimentation of clusters of yeast cells (figure 4*a*), leading to the creation of precisely connected collections of cells called the ‘snowflake’ phenotype (figure 4*b*). This system in turn maintained a characteristic cell cluster size through the selective induction of apoptosis in individual cells. Understanding the dynamics of such evolving systems has drawn efforts from physics-based models [50]; however, an alternative approach lies in using a network approach. The calculation of node betweenness centrality in these yeast structural networks reveals the system to be integrating different layers of physical information into the topology of the ‘snowflake’ by trying to optimize the average path length, as shown by the correlation between apoptotic cells (nodes that are going to disappear and thus break the network) and high node betweenness centrality (figure 4*c*). This approach may be of further value when trying to understand the dynamics and optimization of naturally occurring branching systems like some algae and hyphae in fungi [51].

## 7. Static and dynamic cellular topologies

The type of network analysis used is contingent upon the physical properties of the developmental system being examined. In plants and fungi, adjacent cells are immobilized with respect to one another through shared cell walls [52]. Typical network science measures described above are directly applicable to these systems as intercellular interactions are irreversible.

The physical embedding of cells in space relative to one another in these systems renders the control of the cell cycle and the orientation of cell division planes the central determinants in the construction of multicellular topologies [13,53,54]. In plant systems, the emergence of anisotropic vasculature cells [55] provides a means by which path length may be transcended. Understanding the relationship between space and topology represents a future challenge to uncover how the properties of cell organization are controlled within immotile cellular systems.

In animal systems, cells are capable of moving and growing past one another. The transient nature of these cell interactions makes edges in these networks dynamic, changing the topology of the network. For these systems, alternative approaches to understanding cell organization are required. Temporal network analysis provides one solution, incorporating dynamics into topological analyses [56]. The extent to which cells are motile within organs would have a profound impact on the topological properties of the system. Introducing temporal aspects into spatial networks, such as cell motility, provides the opportunity for individual cells to interact with other cells, regardless of their starting position. In terms of information transport, this would allow specific cells to potentially exert greater influence across the organ. Examples of such behaviour can be found in another class of systems, those displaying collective intelligence, sometimes referred to as ‘fluid neural networks’. Here, each individual unit (an ant, bee or fish) contributes to the final computation of the system, some having a higher impact on the whole by virtue of their enhanced mobility, with interesting implications for engineering [57].

This approach may also be used to study the spatial dynamics within microbial communities where cells are motile. The structural features of these societies may play an important functional role in light of the global communication taking place across colonies [58], and the relationships between different species in cross-feeding contexts [59,60].

## 8. Extraction and annotation of cell interaction networks

Central to cellular interaction mapping is the need to perform imaging, and the computational analysis of these data. Advances in sample preparation [61–63], image acquisition [64] and image analysis [65,66] have facilitated the construction of cellular resolution connectomes.

The use of fixed tissue combined with optical clearing techniques and fluorescence has provided a step change in rapid and accurate acquisition. These approaches enable the deep and high-resolution imaging of optically heterogeneous organs at depths not possible previously [62,67,68]. This is particularly important, as both accurate and complete connectomes are required for their meaningful analysis at cellular resolution. The application of expansion microscopy provides further improvement in accuracy, providing super-resolution imaging of fixed samples at low laser intensities [61]. The preservation of fluorescent proteins and the ability to repeatedly probe fixed and clarified samples with antibodies and nucleotide probes provides the opportunity to add multidimensional functional annotation to structural networks generated using these methods. The ability to annotate structural networks with multiple rounds of functional information within the same sample represents an advantage over live imaging systems which are constrained by the number of fluorescent reporters which can be resolved, typically three. However, the disadvantage of imaging using fixed tissue is the loss of topological dynamics.

Live fluorescent imaging has also been used to track topological dynamics in organs [66,69]. The key advantage of this approach is the ability to retain a living sample and follow the organizational changes in cells within the tissue. Local interactions between cells mediate the formation of patterns through self-organizing principles in diverse organisms. The rules underlying these processes remain largely unknown due to a lack of data at the appropriate resolution and models capable of quantitatively recapitulating them. Live imaging of organs and quantification of their cellular topological dynamics using network science provide a means to quantify the outputs of self-organizing processes and accurately assess models which aim to recreate these processes. This is a central advantage of live imaging; however, the inability to penetrate deep within optically heterogeneous tissues and the limited number of fluorescent reporters that can be visualized at once [70] remain as persistent limitations.

In both fixed and live cell imaging of organs, the computational analysis of cell associations depends upon the ability to accurately segment cells and capture their contacts in space. To achieve this, cell boundary markers are used to delimit the extent of cell segmentation. This can be achieved through the use of genetically encoded membrane or cell wall markers, or with vital fluorescent stains in the case of live imaging. Nuclear markers are therefore not sufficient for generating accurate connectomes as these fail to capture cell shape.

High-resolution connectomes using serial TEM or serial block face imaging and reconstruction have been extracted in the neuroscience field [71], and algorithms to track neuronal trajectories and associations have been developed [72]. This is particularly useful for the resolution of fine cells such as in nervous systems. Similarly, with other tissues, 3D imaging will provide the necessary data to resolve cellular interfaces, which in turn can be algorithmically used to recover the cellular connectomes. Furthermore, improving imaging will increase opportunities for functional annotation of these networks, providing further dimensions to characterize using network science.

Annotation of cell types is also necessary to discriminate topological classes and understand relationships between components of the system. This has been developed previously in *C. elegans* and for radially symmetric organs in plants, through a combination of positional and topological information of cell arrangements [73,74].

## 9. Structural network analyses

The topological analysis of cellular structural networks has been performed in a limited number of instances, and each have provided unique insight into the biological systems examined. These are discussed below and summarized in table 1, along with the strengths and weaknesses of these datasets.

**Table 1.** List of existing cellular structural networks in diverse biological systems.

species	organ	advantages	disadvantages	references
*C. elegans*	nervous system	complete connectome	no spatial information only one cell type	[19]
*Drosophila*	wing disc	contains spatial information live image/dynamic	incomplete/not whole organ	[31,75,76]
*Cucumis* (cucumber)	shoot apical meristem	contains spatial information live image/dynamic	incomplete/not whole organ epithelium only	[32]
*Arabidopsis*	developing embryo	complete connectome contains spatial information	fixed tissue/static images	[77]
*Arabidopsis*, foxglove, poppy	hypocotyl (embryonic plant stem)	complete connectome contains spatial information	fixed tissue/static images	[78]

Neuroscience has been leading the way with cellular interaction network analyses, starting with the complete connectome of *C. elegans* [19]. This seminal dataset has provided the first example of the utility of mapping global cellular interactions in a complex organism. The examination of the higher-order properties across the *C. elegans* nervous system has helped uncover neuronal circuits controlling worm movement [20], temperature sensing [79] and egg-laying behaviour [80]. This directed network is indeed complete, though it only represents a fraction of the cells present in this organism, limiting its utility to the study of nervous system function. It also does not capture the spatial positioning of cells within 3D space.

Following from this work, more ambitious projects seeking to bridge the structure–function relationship in more complex brains, including mouse and humans, are underway [81–83]. These studies hold tremendous scientific and medical potential leading to enhanced understanding of nervous system function and disease.

Developmental studies of structural network properties have also begun to be undertaken. Cell organization has also been microscopically examined in planar systems including the developing *Drosophila* wing disc. Using live cell imaging, local connectivity or degree (number of immediate neighbours) revealed an ability to discriminate cell organization between organs, species, stages of development and genetic backgrounds [75].

Other studies examining the polygonal shape of epithelial cells, a proxy for cell degree in these tessellated tissues, found the number of neighbours a cell has to be tightly regulated in animal and plant epithelia [31]. This work implicated the presence of a mechanism regulating local cell organization across kingdoms. Further work suggested this to be due to a cleavage plane bias during cell division which promotes cells having a regulated number of local neighbours in both plants and animals [32]. More recently, this view has been challenged through the reporting of diverse degree frequencies in tissues, which is related to the balance of cell size and distribution of forces within tissues [76].

In plants, the role of cellular connectivity during embryogenesis has also been explored previously [77,78]. Imaging using confocal microscopy and segmentation of individual cells has enabled the properties of global cellular connectivity to be explored in whole organs. This was first performed in developing *Arabidopsis* embryos at the 16-cell stage [77]. By comparing each the wild-type and transgenic embryo, the role of the transcriptional responses mediated by the hormone auxin was found to impact local cellular connectivity.

The exploration of the higher-order properties of global cellular organization in whole organs has been performed by topologically analysing complete cellular resolution connectomes of the plant hypocotyl (of the embryonic stem) [78]. Path length analysis using betweenness centrality revealed the presence of previously undescribed optimized conduits in the non-hair-forming (atrichoblast) cells of the epidermis of this organ. The preferential movement of exogenously applied fluorescent molecules along the length of the epidermis specifically within this cell type was predicted at single-cell resolution following a high betweenness principle [78]. The passive bulk flow of molecules through complex cellular arrangements in plants may therefore be predicted at single-cell resolution by understanding the higher-order properties of global cellular organization.

This work potentially bridges a structure–function relationship in the patterning of epidermal cells in plants [8]. Two cell types are present including the hair-forming cells (trichoblasts), which acquire nutrients from the environment, and the non-hair-forming (atrichoblast) cells, which are adjacent to these [84]. The functional relevance of having two cell types in the epidermis remains unclear, and this structural analysis of epidermal cell patterning may provide an explanation for this. Hair cells acquire solutes from the soil, and pass these onto the neighbouring non-hair cells for transport up the stem following observation with applied fluorescent molecules [78]. In this way, hair cells can maintain low intracellular solute concentrations, facilitating further nutrient uptake, while molecules are moved along transport-optimized non-hair cell files. This implicates a division of labour between these cell types for nutrient uptake and movement.

In each of these instances, novel insights into the biological system in question were derived by investigating the structural connectivity between cells in organs. The exploration of these structural properties combined with their functional annotation in diverse biological contexts provides a promising and quantitative approach towards understanding developmental processes.

## 10. Functional networks

Structural networks provide the templates upon which genetic networks operate, and where functional information can flow across multicellular assemblies. Functional annotation of structural templates can be achieved through the localization of genes, proteins, transporters and metabolites within single cells of imaged organs. This may be established microscopically through the use of reporter constructs or genetically encoded biosensors.

For two cells to communicate, they must be in physical contact with one another. Whether or not information is indeed being passed from one to the next requires additional functional annotation by experimentation. In a functional context, if two cells are physically associated but not exchanging information, then an edge may be considered to not be present.

The mechanism(s) underlying information exchange between cells differs across kingdoms. Animals cells have gap junctions, plants plasmodesmata and fungi septum pores. Each of these enable the movement of instructive molecules between cells in multicellular assemblies. The size of the physical interactions between adjacent cells plays a role in the capacity for information to be exchanged in light of this being a physical process. Shared intercellular interface size may therefore be considered as one possible edge weighting in the context of a structural network [78].

Establishing functional interactions between cells has been approached previously in diverse contexts. The field of neuroscience has developed coarse-grained models of human brains and measured the flow of information using fMRI data, which are mapped onto a structural region-based template [85,86]. Higher-resolution imaging approaches enable the firing of individual neurons to be visualized using microscopy [87].

In plant science, the mobile hormone auxin, which is central to pattern formation and organ homeostasis, has been studied extensively [88]. Membrane-localized efflux pumps (PIN proteins) and uptake transporters (AUX/LAX proteins) mediate the cell-to-cell movement of auxin across plant organs. The development of fluorescently tagged PIN auxin transporters [89] has enabled the polar localization of these proteins to be identified and the inference of intercellular hormone movement across organs. Mathematical approaches have been applied to understand and predict how auxin gradients are established using this combination of auxin transporter localizations and multicellular templates upon which these act [90,91].

The development of novel imaging techniques involving tissue clarification and multiple rounds of 3D immunolocalization of proteins promises to transform the capacity for the functional annotation of multicellular structural templates with the ability to integrate multiple dimensions of functional annotation within the same sample in the future [62,92,93].

While informative, the abundance of transporter proteins does not strictly correlate with molecular movement between cells. In this regard, a more direct approach to examining actual transport capacity and rates is required. An example of how this may be achieved is activating photoactivatable molecules (caged molecules or photoactivatable fluorescent proteins) in individual cells, and quantifying their movement across the system. The development of additional approaches to functionally annotate edge weights (measured transport) in multicellular networks represents a key obstacle to overcome before a comprehensive systems-level understanding of organ function can be achieved.

## 11. Further potential for developmental connectomics

The capture of complete organism-wide connectomes and their functional annotation provides a means to address fundamental questions that cannot be examined otherwise, namely the exploration of the higher-order properties of complex multicellular assemblies. Uncovering these structures and their properties can reveal selective pressure on particular architectures and features over the course of evolution.

Non-optimized arrangements may also be explored. In many organisms, genes which mediate the correct arrangement of cells have been identified, and viable individuals carrying mutations in these genes can be grown in the laboratory. The topological analysis of these alternative cell arrangements can establish how features of cell topology are genetically encoded, and potentially how these have been optimized. In many instances, conflicts between cells manifest at a mechanical level [94], and a role for cell organization in the control of organ morphogenesis has been established previously [95]. The analysis of cell organization and the ability for this to identify cancerous tissue have been reported previously [96], suggesting this analysis may also have diagnostic value.

By discretizing, analysing and understanding the properties of complex cellular arrangements within the network science framework, it becomes possible to understand what cellular architectures have been selected for and persisted in the natural world. Understanding these extant topologies, and revealing the principles underlying the drive to complexity may also pave the way for the prediction of future organ designs through morphospace analyses [9]. Such approaches combined with synthetic biology [97,98] can provide a discrete framework for the rational re-engineering of complex multicellular systems [99]. In this way, organisms with novel functions may be generated following known structure–function principles, through morphogenetic engineering [100].

## Data accessibility

This article has no additional data.
